# Role of Circulating Tumor DNA in Gastrointestinal Cancers: Update From Abstracts and Sessions at ASCO 2018

**DOI:** 10.3389/fonc.2019.00358

**Published:** 2019-05-08

**Authors:** Faisal Shahjehan, Saivaishnavi Kamatham, Pashtoon Murtaza Kasi

**Affiliations:** ^1^Division of Hematology, Oncology, Department of Internal Medicine, Mayo Clinic, Jacksonville, FL, United States; ^2^Division of Hematology, Oncology, University of Iowa Health Care, Iowa City, IA, United States

**Keywords:** circulating tumor DNA, ctDNA, liquid biopsy, ASCO, gastrointestinal cancer, colorectal cancer

## Abstract

**Background:** The promising aspect of circulating tumor DNA (ctDNA) is its rapid turnaround and non-invasive nature. According to the American Society of Clinical Oncology (ASCO) and College of American Pathologists joint ctDNA review published in March 2018, there is not sufficient evidence to support the use of ctDNA in practice for GI cancers. However, there were numerous studies presented at ASCO Annual Meeting supporting its value. We aimed to summarize on its role in the management of gastrointestinal cancers based on the studies presented recently, and future directions.

**Methods:** We limited our search to keywords “ctDNA,” “circulating tumor DNA,” “cell-free DNA (cfDNA)” and/or “liquid biopsy,” at the 2018 ASCO Annual Meeting library abstracts and presentations.

**Results:** There were 35 studies that revolved around ctDNA as a diagnostic tool, prognostic marker and/or a measure of tumor heterogeneity in gastrointestinal cancers. Depending on the assay used, the results of several studies showed that ctDNA was able to identify relevant mutations or fusions including *RAS, HER2/Neu, BRAF, MET, BRCA2, APC, TP53, ALK, ROS1, PTEN*, and *NF1*. The prognosis in terms of tumor mutation burden, objective response rate, metastasis and survival were also estimated by various studies based on ctDNA. The findings showed that higher baseline ctDNA levels and/or increased number of mutations detected in ctDNA were associated with poor survival and multi-site metastasis. Right-sided colon cancer was associated with higher number of mutations in ctDNA than left-sided colon and rectal cancers. Similarly, tubular adenocarcinoma subtype of gastric cancer was more likely to have higher ctDNA levels than signet-ring cell subtype. The feasibility of assessing response to therapy and residual metastatic disease by using ctDNA which was otherwise not detected on imaging was also presented.

**Conclusions:** The studies presented at ASCO 2018 report on the many ways ctDNA is of value in patients with gastrointestinal malignancies. Experts and discussants at the meeting argued that this may well indeed be ready for prime time for certain GI malignancies including colorectal cancers, especially in the metastatic setting. These findings alongside ongoing studies showing its feasibility into practice would likely lead to revision of the current guidelines for metastatic GI cancers.

## Introduction

Circulating tumor DNA (ctDNA), considered as cancer biomarker, is the free DNA found predominantly in plasma and derived from tumor cells. Analysis of ctDNA, also referred to as “liquid biopsy,” is a non-invasive and cheaper technique allowing for serial oncologic assessments, though several controversies exist. In March 2018, American Society of Clinical Oncology (ASCO) and the College of American Pathologists published a joint review on ctDNA. They reported on the value in terms of clinical utility and validity for few types of late stage cancers, but not for the majority of late stage cancers and not at all for early stage cancers (1). The consensus statement therefore narrated that routine ctDNA testing is not yet ready to be considered as an integral part of the management of cancers. Since the publication of this review in March 2018, the 54th ASCO Annual Meeting was convened at Chicago, Illinois on June 1–5, 2018. The research abstracts and the discussions, however, at ASCO conference argued otherwise.

In this article we aim to highlight the recent key updates and advances related to ctDNA presented at 2018 ASCO Annual Meeting that was held after the consensus statement. A literature search of 2018 ASCO Annual Meeting library, by using the key words “ctDNA,” “circulating tumor DNA” “cell-free DNA (cfDNA)” or “liquid biopsy” was done; and the most relevant abstracts pertaining to GI malignancies were the ones selected to be summarized/included in this review.

## ctDNA as a Diagnostic Tool

Tissue biopsy remains the gold standard test for the diagnosis of cancers. Furthermore, genetic testing done on tissue is what is used to find actionable genes or aberrations. The yield depending on site and procedure often is enough to make the diagnosis but not enough to run additional genetic tests. There were an array of studies reporting on the feasibility and specificity of liquid biopsies aiding and/or corroborating findings noted on tissue biopsies in gastrointestinal cancers including colorectal cancers (CRC) and non-colorectal cancers. Hu et al. investigated the role of ctDNA in CRC patients and reported that the number of DNA mutations detected in tissue biopsy correspond with that found in liquid biopsy (2). Huang et al. did a study on 30 CRC patients who underwent surgery and presented that the majority of patients (83%) had at least one mutation detected in both tissue biopsy as well as liquid biopsy (3). A study conducted on anal cancer patients demonstrated the ability of ctDNA in terms of sensitivity (89%) to help diagnose the cancerous disease (4). An Australian study reported the detection ctDNA in 62.2% pre-operative and 37.1% post-operative plasma samples of patients with early stage pancreatic cancer (5). Mody et al. conducted a study on 104 cholangiocarcinoma patients and reported that at least one gene mutation was identified in ctDNA sample in 77% of patients (median number of mutations per patient = 3; range = 1–15) (6). Another study revealed high mutational concordance between liquid and tissue biopsy for biliary tract cancer (74%) and intrahepatic type (92%) (7). In another study of gastroesophageal cancer patients, researchers were able to detect at least one mutation (median number of mutations per patient = 2; range = 0–15) in ctDNA in 66% of patients (8).

Many clinical trials, mostly ongoing, have demonstrated that the mutations in oncologic genes responsible for carcinogenesis can be identified in ctDNA. These ctDNA mutations correlate well with tissue biopsy results. In the REVERCE phase II trial studying early exposure to regorafenib vs. anti-EGFR in patients with CRC, Tsuji et al. identified mutations in several genes including *RAS, BRAF, EGFR, HER2*, and *MET* using ctDNA samples of patients with metastatic CRC (Clinical trial: UMIN000011294) (9). What was interesting was that the arrays of mutations acquired were different depending on the sequence of therapy (Regorafenib anti-EGFR vs. anti-EGFR Regorafenib). Similarly, in the HERACLES study, a phase II trial of trastuzumab and lepatinib in *HER-2* positive metastatic CRC, researchers were able to correctly identify the *HER-2* amplifications in 96% of samples using ctDNA (10). In another study, researchers reported that *HER-2 (ERBB2) amplifications* were identified in 61% of gastroesophageal cancer patients using ctDNA samples (11). Iqbal et al. detected a variety of mutations in different genes including *HER2, BRCA2, TP53, APC, ROS1, PTEN, KRAS, CCEN1, GNAS, NF1, CTNNB1, PIK3CA*, and *ARID1A* using ctDNA in gastroesophageal adenocarcinoma patients (12). Jia et al. demonstrated the practicality of employing cfDNA for the detection MET amplification in patients with RAS wild-type metastatic CRC (Clinical trial: NCT02008383). They enrolled the patients in two groups i.e., one receiving cabozantinib plus panitumumab and the second receiving cabozantinib alone; and found detectable cfDNA levels and MET amplification in 98 and 18% of patients, respectively (13). In WJOG7112G study, Sukawa et al. studied gastric or gastroesophageal cancer patients who had disease progression despite receiving chemotherapy, and identified HER2 amplifications mutations in ctDNA in 60% of patients (14).

In summary, multiple studies show that GI malignancies in general shed DNA that can be detected and the current technologies available corroborate and correlate well with tissue based genetic testing.

## ctDNA as a Prognostic Biomarker

### Prediction of Response to Therapy

The behavior of cancer in response to therapy can be predicted by determining the type and number of ctDNA mutations. The research presented at ASCO conference does establish ctDNA as an independent prognostic marker in many cancers. Zhang et al. investigated ctDNA of 43 esophageal squamous cell carcinoma patients, and reported the role of ctDNA in predicting response to therapy. Their results showed that the patients who did not respond to neoadjuvant chemotherapy were associated with higher driver gene molecular mutation burden compared to those who responded well (*p* < 0.01) (15). Yang et al. studied 88 rectal cancer patients and reported that ctDNA levels became undetectable during neoadjuvant chemoradiotherapy in 65.5% of the patients which were congruous with the imaging and histological changes (16). Another study examined various driver mutational genes detected by ctDNA in CRC patients and reported a noticeable reduction in tumor mutation burden following surgery (3).

There were also studies that reported on ctDNA as a biomarker of the efficacy of specific chemotherapeutic agents in different cancers. Catenacci et al. investigated the response of margetuximab plus pembrolizumab in ERBB2-positive gastroesophageal cancer patients, and demonstrated that the response to therapy was predicted based on ctDNA sample results. They calculated objective response rate and disease control rate using ctDNA which were 57 and 86%, respectively, for their cohort. They also reported that a lot of cancer patients lost their ERBB2 amplifications as detected by ctDNA after receiving trastuzumab (11). Chen et al. conducted a phase II study in China and assessed the clinical rationale of apatinib in chemotherapy-refractory metastatic CRC patients (Clinical trial: NCT03190616). They reported that tumor mutation burden calculated by ctDNA is the main factor determining prognosis (17). In HERACLES study discussed earlier, the researchers reported that ctDNA precisely anticipated the response to HER-2 receptor inhibitor therapy in HER2-positive CRC (10).

**Figure 1:**
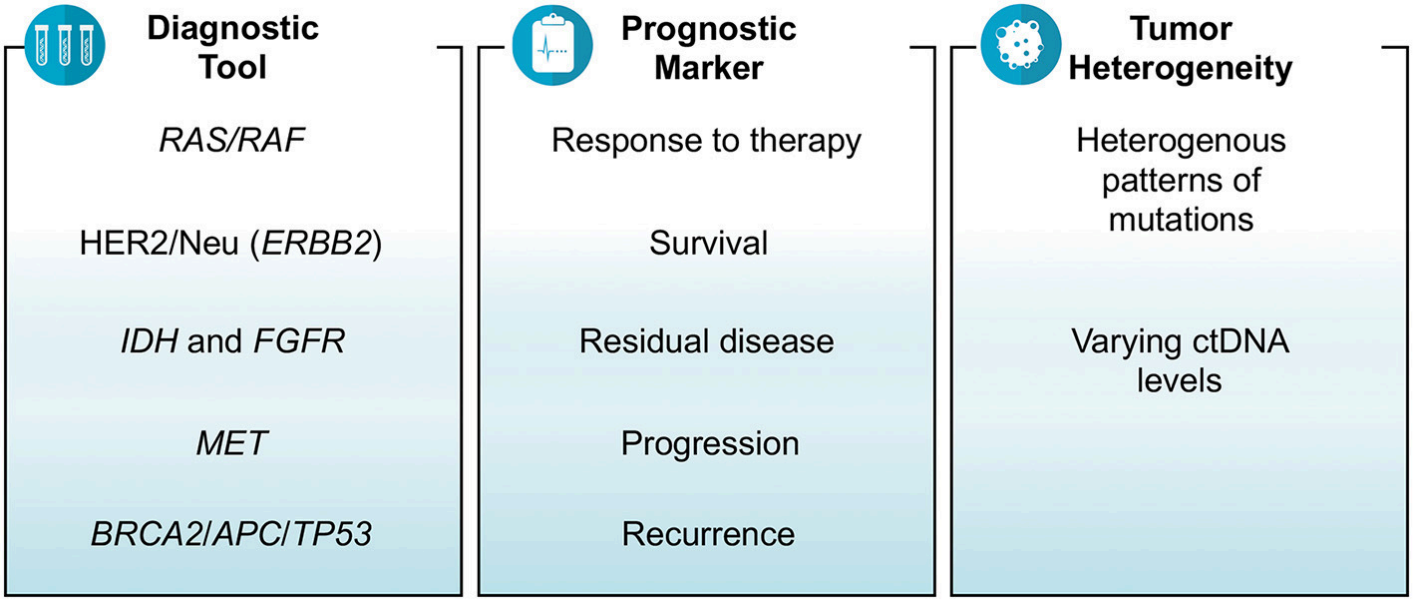
Summary of studies reporting on use of ctDNA in GI malignancies at ASCO 2018.

The conclusions one can draw from these studies are that in general if there is a decrease in the variant allele fraction or the number of mutations (or lack of detection of any ctDNA) after receiving therapy, it affirms an improvement in terms of reduction in tumor size. Studies noting a decline in ctDNA as early as 2 weeks could predict response to therapy months later on imaging studies. Unrelated, but utilizing ctDNA testing is also helpful in patients having pseudoprogression in their imaging on immunotherapy. These studies presented at ASCO conference do strongly support ctDNA as a reliable cancer biomarker and favor its use in the management of cancer patients in prognosticating and as a dynamic early biomarker predicting response to therapy (Figure 1).

### Prediction of Survival

Researchers estimated and reported survival rates of CRC based on the findings of ctDNA. Siveke et al. studied the role of ctDNA in determining the prognosis of metastatic CRC and reported that high baseline ctDNA is associated with multi-site metastasis, increased levels of carcinoembryonic antigen, propensity of having right-sided origin and poor overall performance compared to low baseline ctDNA. They further demonstrated that baseline ctDNA is the predictor of progression free survival (HR = 2.01; 95% CI: 1.25–3.22, *p* = 0.0033) (18). Poulsen et al. showed the association of ctDNA mutations and overall survival in patients with metastatic CRC. They reported that the patients who received Sym004 (anti-EGFR antibody mixture) and were negative for *RAS* and *BRAF V600E* mutation in ctDNA had an overall survival advantage of 3.5 months compared to those who received standard therapy (HR: 0.71; *p* = 0.134). They further estimated the overall survival of patients who were negative for EGFR mutations in addition to *RAS* and *BRAF V600E* mutation in ctDNA (HR: 0.59; *p* = 0.044) (19). Kehagias et al. investigated 141 patients with advanced CRC in a multicentric trial, and measured cfDNA levels at baseline and at day 14 after starting regorafenib (Clinical trial: NCT01929616). The patients having cfDNA levels ≥1 μg/ml at day 14 were associated with poor progression-free (HR: 2.50, 95% CI: 1.73–3.63) and overall survival (HR: 3.83, 95% CI: 2.52–5.71) compared to those having levels <1 μg/ml (20). A Japanese study showed similar results and reported that the finding of *KRAS* mutated ctDNA in post-chemotherapy metastatic CRC patients is associated with reduced progression free survival (21). The high levels of ctDNA and high tumor mutation burden correlated with poor survival rates of CRC.

Survival of non-colorectal cancers including esophageal and biliary tract cancers were also estimated based on the numbers of mutations detected in ctDNA. A study of esophageal squamous cell carcinoma patients who underwent radiation therapy reported ctDNA as one of the main factors determining prognosis. Their results revealed that the patients who had mutations in ctDNA after undergoing radiation therapy were associated with poor overall survival (*p* = 0.005) and a trend of a decrease in disease free survival (*p* = 0.068) compared to those who did not have any mutations in ctDNA (22). A German study showed the association of variant allele frequency detected by ctDNA and progression-free survival of intrahepatic biliary tract cancer (Spearman, *r* = −0.5878, *p* = 0.0288) (7).

### Detection of Residual Disease, Progression, and Recurrence

Cancer patients are evaluated after completion of therapy to make sure there is no possible tumor left behind. Imaging and/or tumor markers are usually checked to rule out any residual disease. The feasibility of using ctDNA as a biomarker of residual disease was reported in various studies at ASCO 2018. Tie et al. conducted a study on 95 stage III colon cancer patients and reported that the response to therapy and the residual metastatic disease which is otherwise not identified on imaging can be detected by ctDNA. Their results showed that the finding of positive ctDNA post-surgery (HR: 3.52; *p* = 0.004) and post-chemotherapy (HR: 7.14; *p* <0.001) is associated with poor recurrence free survival (23). Murray et al. did a prospective study on 172 post-surgery CRC patients, and demonstrated that ctDNA is a marker of residual disease and recurrence (Clinical trial: 12611000318987). Their results showed that the patients who were positive for ctDNA after surgery were at an increased risk of recurrence (HR: 3.8, 95% CI: 1.5–9.5) (24).

Furthermore, studies have shown the reliability of the use of ctDNA in ascertaining the disease progression in terms of metastasis. A study conducted on CRC patients demonstrated the association of post-surgery detection of ctDNA mutations and increased risk of disease progression. The researchers found that higher proportion of ctDNA mutation positive patients (27.8%) experienced disease progression compared to those who were ctDNA mutation negative (4.4%) after surgery (25). Another study showed the reliability of ctDNA in predicting the response to regorafenib or TAS-102 in patients with metastatic CRC using different PCR based methods (26). Cabel et al. conducted a study on 36 anal cancer patients in France and reported that post-chemoradiotherapy detection of ctDNA is associated with worse outcomes. Their results showed that 17% of the patients had metastatic relapse, and these were the only patients who were positive for ctDNA after receiving chemoradiotherapy (4). Parseghian et al. demonstrated the usefulness of ctDNA in monitoring the decay of clones which were resistant to anti-EGFR agents in metastatic CRC. They calculated the median relative mutant allele fraction (10.5 vs. 10.6%) and the decaying half-life of clones (3.4 vs. 6.9 months) for *RAS* and *EGFR*, respectively, while on anti-EGFR therapy (27). Acquisitions of these mutations are known mechanisms of secondary resistance in patients with metastatic CRC. What is indeed intriguing is that these clones can be lost over time allowing for potentially “rechallenging” some of these therapies leading to development of multiple trials employing this strategy.

**Figure 2:**
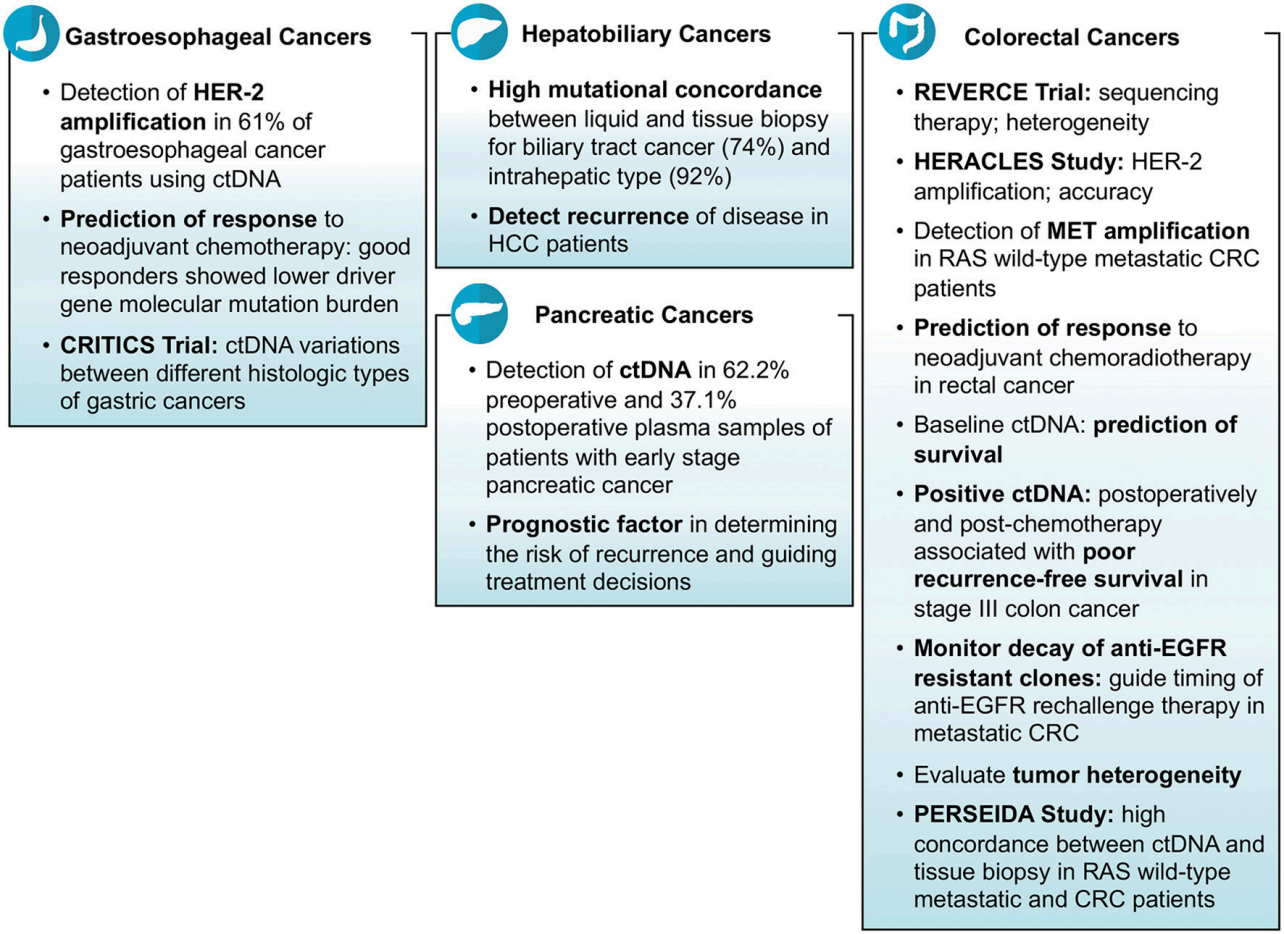
Findings of ASCO 2018 studies about the use of liquid biopsy in gastrointestinal cancers.

Finally, recurrence of cancers is a common phenomenon and ctDNA was noted in multiple studies to be helpful in predicting the risk of recurrence after being treated for the primary cancer (Figure 2). Murray et al. studied the association of ctDNA and recurrence of CRC, and reported that post-surgery detection of ctDNA is associated with an increased risk of recurrence (HR = 3.8; *p* = 0.004) (Clinical trial: 12611000318987) (28). A Chinese study, conducted on hepatocellular carcinoma patients who underwent transcatheter arterial chemoembolization, reported that high mutational burden of 10 genes identified in ctDNA might be associated with the recurrence of the disease. The 10 genes detected in their study were *NRAS, BRAF, PIK3CA, KRAS, ARID1A, AXIN1, ARID2, TERT, TP53*, and *CTNNB1* (29). Lee et al. demonstrated that ctDNA is an indicator of prognosis in terms of determining the risk of recurrence and guiding treatment decisions in patients with early stage pancreatic cancer (Clinical trial: ACTRN12612000763842). Their analysis showed that detection of ctDNA both before and after surgery is associated with poor recurrence free and overall survival; and 100% of patients showed recurrence who had measurable ctDNA after surgery despite of being on adjuvant chemotherapy (5).

## Tumor Heterogeneity and ctDNA

Gastrointestinal cancers including CRC are heterogeneous in nature in terms of histology, presenting symptoms, progression, response to therapy, and outcomes that can lead to a difference in the management of various types of cancers (30). Tumor heterogeneity can be evaluated by measuring the wide-ranging levels of ctDNA and the identification of diverse patterns of mutations. A study reported that right-sided colon cancer is associated with an increased number of mutations in ctDNA than left-sided colon and rectal cancers (2). Another study demonstrated higher median cfDNA levels (14.2 vs. 8.94 ng/ml) and higher mutation concordance rate identified via liquid and tissue biopsies (94.7 vs. 50%) for colon cancer patients than rectal cancer patients (31). Similarly in another study, researchers identified a heterogeneous pattern of genomic alterations in ctDNA of patients with metastatic CRC including mutations in *BRAF, KRAS, NRAS, MAP2K1, PIK3CA, ERBB2, MET*, and *EGFR* genes (19). A Chinese study on patients with rectal cancer indicated that the most common mutations found in ctDNA were of *TP53, APC, and KRAS* genes (16). A study of cholangiocarcinoma patients reported the detection of mutations in ctDNA in a variety of genes including *TP53, KRAS, FGFR2, ARID1A, APC, PIK3CA, BRAF, CCND1, CCND2, CCNE1, CDK4, CDK6, EGFR, ERBB2, FGFR1, MET, MYC*, and *PDGFRA* (6). The same study revealed the finding of FGFR2 fusions and the actionable mutations in 3 and 61% of patients, respectively (6). Clifton et al. did a study for the identification of actionable gene fusions in CRC and reported that the main fusions detected in ctDNA samples were *RET, FGFR3, ALK, NTRK1, ROS1*, and *FGFR2* (32).

Several clinical trials have demonstrated the role of ctDNA in detecting tumor heterogeneity of different gastrointestinal cancers. In CRITICS phase III trial, Leal et al. investigated cfDNA of stage Ib-IVa resectable gastric cancer patients (*n* = 115) and showed that the level of ctDNA varies between different histologic types of cancer (Clinical trial: NCT00407186). They found higher ctDNA levels for tubular adenocarcinoma (mutant allele fractions: 0.25%) than signet-ring cell subtype (mutant allele fractions: 0.16%) of gastric cancer (33). Yaung et al. demonstrated the ability of ctDNA, by using next-generation sequencing based methods, to detect tumor heterogeneity in a clinical trial (STEAM) evaluating the outcomes of FOLFOXIRI-bevacizumab vs. FOLFOX-bevacizumab as first-line treatment of metastatic CRC (Clinical trial: NCT01765582). Their results showed that high mutant-allele tumor heterogeneity is associated with poor outcomes (34). Okamura et al. examined the ctDNA of gastroesophageal carcinoma patients (*n* = 55) and found different arrays of genomic alterations (Clinical trial: NCT02478931). The most frequent mutations found in their study were *TP53, PIK3CA, ERBB2* and *KRAS* in 50.9, 16.4, 14.5, and 14.5% of patients, respectively (8). In another clinical trial, the mutated genes on ctDNA in patients with advanced CRC were found to be *APC, TP53, KRAS*, and *PI3KCA* in 73, 72, 66, and 23% of ctDNA samples, respectively (20). PERSEIDA study which is an ongoing trial in Spain evaluated the concordance in mutation profile between ctDNA and tissue biopsy. The researchers analyzed the data of tissue biopsy-proven *RAS* wild type metastatic CRC patients (*n* = 119) from 20 centers who were later assessed with liquid biopsy, and reported that there was high concordance between the two but new *RAS* mutations were also detected in ctDNA majority of which were at low mutant allele fraction limit (Clinical trial: NCT02792478) (35).

## Future Directions and Conclusion

While the joint statement issued earlier in March 2018 was reasonable, the array of studies presented and discussed at ASCO 2018 argue more in favor of integrating ctDNA in practice for many gastrointestinal malignancies. For some e.g., CRC, gastroesophageal cancers as well as biliary duct cancers, it is helpful in not only identifying relevant actionable mutations but also in helping identifying secondary mechanisms of resistance. Furthermore, it helps in guiding therapy, as the appropriate chemotherapy drugs can be added to the treatment regimen depending on the emerging clones of resistance detected on ctDNA testing. It also aids in monitoring the response to treatment and predicting survival depending on the level of ctDNA detection in serial analyses. While the technology and the methods may not be ready for use in all stages in all cancers, for patients with metastatic GI cancers, particularly CRC, we would argue similar to discussants at colorectal cancer sessions at ASCO that this is indeed ready for primetime because of its feasibility, rapid turn around and non-invasive nature allowing for serial oncologic assessments. Reevaluation of the literature is needed which could result in an update in the current guidelines for use of ctDNA particularly in late stage gastrointestinal cancers.

## Author Contributions

All authors listed have made a substantial, direct and intellectual contribution to the work, and approved it for publication.

### Conflict of Interest Statement

PK has provided advisory board consultancy to Taiho in January 2017 (to institution) and Ipsen in June 2018 (to institution). The remaining authors declare that the research was conducted in the absence of any commercial or financial relationships that could be construed as a potential conflict of interest.
